# Comparative efficacy evaluation of inverted internal limiting membrane flap technique and internal limiting membrane peeling in large macular holes: a systematic review and meta-analysis

**DOI:** 10.1186/s12886-019-1271-2

**Published:** 2020-01-08

**Authors:** Yu Shen, Xiaoqin Lin, Luyi Zhang, Miaoqin Wu

**Affiliations:** 1 Second Clinical Medicine Faculty, Zhejiang Chinese Medical University, Hangzhou 310053 Zhejiang Province, People’s Republic of China; 2 Department of Ophthalmology, Zhejiang Provincial People’s Hospital, People’s Hospital of Hangzhou Medical college, Hangzhou 310014 Zhejiang Province, People’s Republic of China

**Keywords:** Inverted internal limiting membrane flap technique, Internal limiting membrane peeling, Large macular hole, Meta-analysis

## Abstract

**Background:**

The purpose of this study was to compare the anatomical and visual outcomes of inverted internal limiting membrane (ILM) flap technique and internal limiting membrane peeling in large macular holes (MH).

**Methods:**

Related studies were reviewed by searching electronic databases of Pubmed, Embase, Cochrane Library. We searched for articles that compared inverted ILM flap technique with ILM peeling for large MH (> 400 μm). Double-arm meta-analysis was performed for the primary end point that was the rate of MH closure, and the secondary end point was postoperative visual acuity (VA). Heterogeneity, publication bias, sensitivity analysis and subgroup analysis were conducted to guarantee the statistical power.

**Results:**

This review included eight studies involving 593 eyes, 4 randomized control trials and 4 retrospective studies. After sensitivity analysis for eliminating the heterogeneity of primary outcome, the pooled data showed the rate of MH closure with inverted ILM flap technique group was statistically significantly higher than ILM peeling group (odds ratio (OR) = 3.95, 95% confidence interval (CI) = 1.89 to 8.27; *P* = 0.0003). At the follow-up duration of 3 months, postoperative VA was significantly better in the group of inverted ILM flap than ILM peeling (mean difference (MD) = − 0.16, 95% CI = − 0.23 to 0.09; *P* < 0.00001). However, there was no difference in visual outcomes between the two groups of different surgical treatments at relatively long-term follow-up over 6 months (MD = 0.01, 95% CI = − 0.12 to 0.15; *P* = 0.86).

**Conclusion:**

Vitrectomy with inverted ILM flap technique had a better anatomical outcome than ILM peeling. Flap technique also had a signifcant visual gain in the short term, but the limitations in visual recovery at a longer follow-up was found.

## Background

Macular hole (MH) is an anatomical defect in the fovea of retina that cause severe visual impairment. It was regarded as an untreatable disease in poor prognosis until the first describtion of vitrectomy to treat MH by Kelly and Wendel [1] in 1991. The success rate of MH surgery increased to 98% [2–4] of cases with the introduction of internal limiting membrane (ILM) peeling by Eckardt et al. [5], which was thought to be one of the most effective surgical procedures [6]. However, the anatomical success rate of MH that are larger than 400 μm is less likely to close and has been as low as 40% [7, 8], regardless of whether the ILM has been removed or not during vitrectomy. Michalewska et al. [9] first presented a novel technique of inverted ILM flap for the treatment of large MH, contributing to a relatively high MH closure rates. Recently, a number of clinical studies have suggested that inverted ILM flap technique achieved better anatomical and visual outcomes than ILM peeling [10–15]. However because of lacking appropriate controls, uncontrollable elements, or insufficient samples in most of these studies, the reliable evidences to support such a view were limited. As far as we know, double-arm study to compare anatomical and visual outcomes between these two methods in MH larger than 400 μm have not been systematically reviewed and published. Thus, we conducted a comprehensive meta-analysis to evaluate the efficacy of vitrectomy with inverted ILM flap technique and ILM peeling.

## Methods

### Search strategy

We cautiously searched for studies that used inverted ILM flap technique or ILM peeling to treat MH larger than 400 μm. The Pubmed, Embase, Cochrane Library databases were systematically searched for all articles including relevant prospective and retrospective clinical trials published before December 2018. The terms used for systematic search were:(macular hole OR macula hole OR MH OR macular break OR macular fissure OR retinal perforations OR retinal break OR retinal hole OR retinal tear) AND (inner limiting membrane OR internal limiting membrane OR ILM OR limiting membrane) AND (peeling OR peel OR removal OR IP OR SIP) AND (inverted OR inversion OR invert OR flap OR flap technique OR IF OR IFT). Furthermore, We enlarged retrieval coverage and manually searched reference lists of original studies, gray literatures and records, without language or publication year restrictions.

### Inclusion and exclusion criteria

All abstracts, studies and citations were reviewed and assessed. The inclusion criteria for eligibility were as follows: (1) double-arm studies; (2) studies included cases among patients with MH larger than 400 μm who had been treated with the inverted ILM flap technique or ILM peeling; (3) anatomical hole closure rate and visual acuity (VA) were observed after the treatments; (4) the relevant statistics were provided, such as age, gender, duration of disease, hole size, and follow-up time; (5) prospective randomized control trial or retrospective case series. Exclusion criteria were as follows: (1) non - controlled study; (2) patients with macular retinoschisis, age-related macular degeneration, retinal detachment, or proliferative diabetic retinopathy; (3) treatments with modified inverted ILM flap techniques; (4) short -term follow-up that less than three months; (5) reviews or case reports.

### Data extraction

Data were independently extracted and reviewed from each included study by two reviewers (YS and XQL). Any discrepancy between data extractions were resolved by the discussion or consulted by the expert. The following data were extracted: first author, year of publication, type of trials, country, surgical procedure, number of eyes involved, patient demographics, age, duration of disease, minimum diameter of MH, hole closure rate, preoperative and postoperative VA, and follow-up time.

### Quality of assessment

This review included eight studies: 4 randomized control trials (RCTs) and 4 retrospective case series. The included RCTs were evaluated for quality in accordance with the “risk of bias” tool recommended by the Cochrane Handbook 5.1.0 [16].Seven items were assessed: “random sequence generation,” “allocation concealment,” “blinding of participants and personnel,” “blinding of outcome assessment,” “incomplete outcome data,” “selective reporting,” and “other bias.” According to whether the included studies fully meet the above criteria, we assessed the quality of trials. The methodological quality of each study was assessed based on the Newcastle-Ottawa Scale (NOS) [17] (range, 0 to 9 stars) for quality of case control studies in meta-analysis. Studies were rated in three areas, including selection, comparability and exposure. Scores ≥5 indicated that the quality of research were relatively high. All items were independently assessed by two investigators (YS and XQL), with consensus reached after discussion or expert consultation.

### Statistical analysis

Data analysis was collated and analyzed by Review Manager 5.3 software (RevMan 5.3, The Cochrane Collaboration, Oxford, UK). For the rate of MH closure, odds ratios (ORs) and 95% confidence intervals (Cl) were calculated by using Mantel–Haenszel (M-H) method. To compare the evaluation of VA, the mean difference (MD) of preoperative and postoperative measurements between the two surgical treatments were compared using weighted MD and 95% CI. The sofeware estimated statistical heterogeneity among studies using I^2^ statistic. When I^2^ > 50%, it suggested there was significant heterogeneity. A random-effects model was used for data synthesis in the presence of significant heterogeneity, while a fixed-effects model was used when there was no significant heterogeneity. The results of meta - analysis was visually examined by forest plot, and the potential publication bias was showed by funnel plot. *P* < 0.05 was considered statistically significant.

## Results

### Selection of studies

**Fig. 1:**
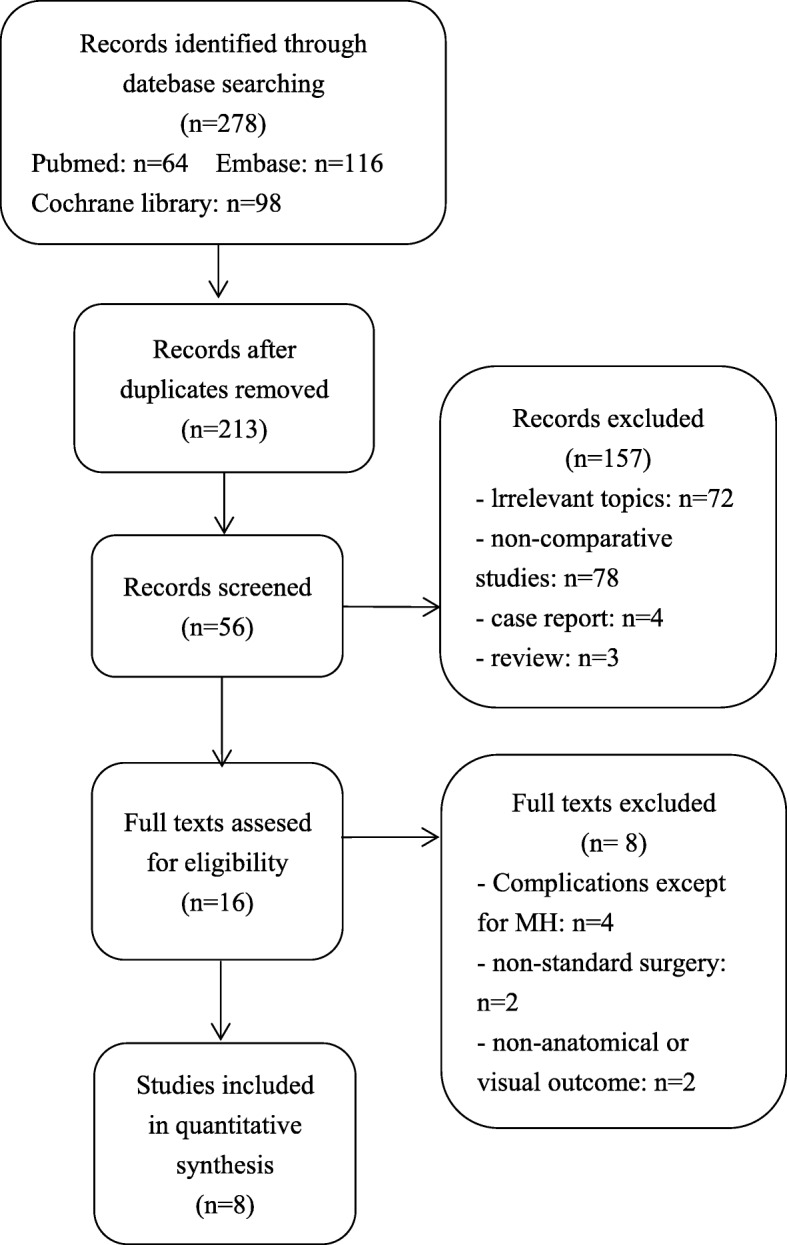
Flow diagram of the study selection process

Totally, 278 articles were initially searched from electronic databases. 206 studies were left for further analysis after duplications, case reports and reviews removed. The titles and abstracts reviewed, and the remaining 16 studies were retrieved for the next review. Finally, after reading carefully of full-texts, We included a total of 8 studies [4, 9–15], 4 RCTs [4, 9–11] and 4 case series [12–15]. Fig. 1. depicts the search process.

### Study characteristics

The main characteristics of the included studies are listed in Table 1. This review included 8 studies, 4 RCTs and 4 case series. It enrolled 578 patients with a total of 593 eyes, including 325 in the ILM peeling group and 268 in the inverted ILM flap group. Their mean age ranged from 59.37 to 69.9 years. The mean duration of symptoms varied from 3.06 to 20 months, and the average minimum diameter of MH ranged from 493.8 to 803.33 μm. The shortest follow-up duration was 3 months in 3 studies [4, 10, 12], the follow-up duration ≥6 months in 5 studies [9, 11, 13–15].

**Table 1:**
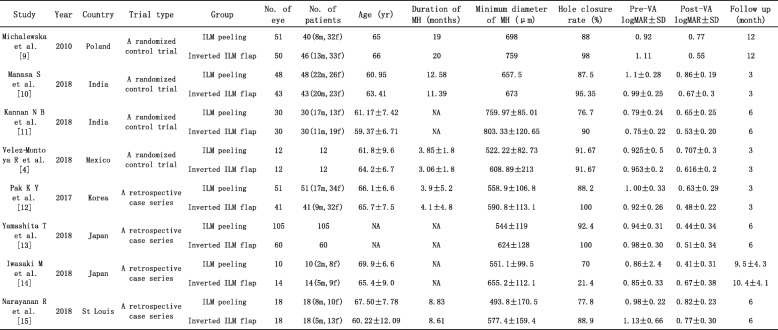
Study characteristics of eligible clinical studies

*ILM* internal limiting membrane, *m* male, *f* female, *NA* not available, *MH* macular hole, VA visual acuity, *logMAR* logarithm of minimal angle of resolution, *SD* standard deviation

### Quality assessment

We assessed the quality of the 4 RCTs using cochrane collaboration’s tool. Methodological quality of eligible trials was moderate to good, and is explained comprehensively in Table 2. The included RCTs had low risk of bias in general, which only had 1 or 2 items with” unknown risk of bias”, except the study reported by Velez-Montoya et al. [4] that had low risk of bias in all assessing criteria and was assessed as a high-quality trial. Double-blinding and reporting outcomes completely were not mentioned in parts of trials, which suggested that the results might be affected slightly by performance bias and selective bias. Of the 4 case series included for quality assessment based on NOS, all studies met 6 or more stars out of 9. All participants included in the case series measured the conditions in a standard and reliable manner. The selection of cases and comparability between case and control trials were clearly reported. The qualitative assessment of case series is presented in Table 3.

**Table 2:**
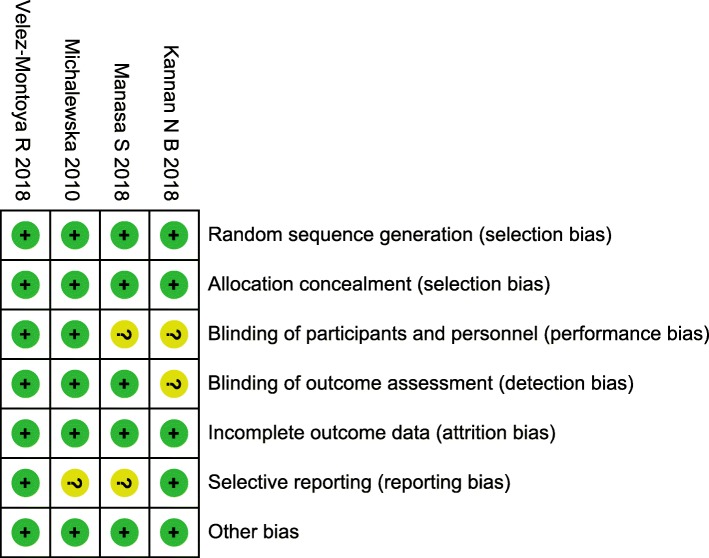
Summary of “risk of bias” assessment

The Cochrane “risk of bias” tool was used for quality assessment. Green for “yes” and yellow for “unclear

**Table 3:**
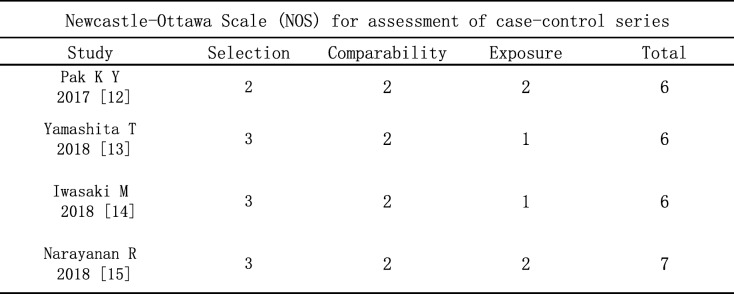
Newcastle-Ottawa Scale table

### The rate of macular hole closure rate analysis

**Fig. 2:**
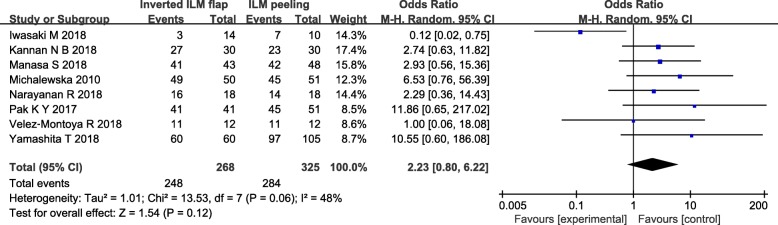
Forest plots of MH closure rate between inverted ILM flap and ILM peeling group

All the included studies reported the rate of MH closure after treated with ILM peeling or inverted ILM flap technique. The rate of MH closure was 92.5% (248/268 eyes) in the inverted ILM flap technique group and 87.4% (284/325 eyes) in the ILM peeling group. These results suggested that the MH closure rate wasn’t significantly different between two groups (OR = 2.23, 95% CI = 0.80 to 6.22; *P* = 0.12; Fig. 2). However, there revealed a moderate heterogeneity between these studies (heterogeneity I^2^ = 48%).

**Fig. 3:**
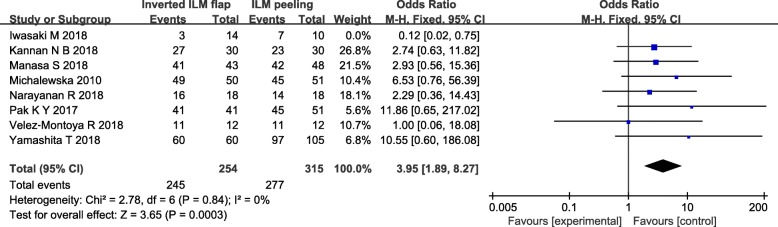
Forest plots of MH closure rate between inverted ILM flap and ILM peeling group after removing Iwasaki’s study

For further clarifying the source of heterogeneity, we performed sensitivity analysis to determine whether existing statistics were stable. Sensitivity analysis were conducted by deleting each included study step by step, and revealed that the trial reported by Iwasaki et al. [14] produce the significantly affect the pooled result, which was considered to be the source heterogeneity. When this trial was removed, there was no statistical heterogeneity between all other studies (heterogeneity I^2^ = 0%). The meta-analysis of pooled data showed MH closure with inverted ILM flap technique group was statistically significantly higher than ILM peeling group (OR = 3.95, 95% CI = 1.89 to 8.27; *P* = 0.0003; Fig. 3).

### Preoperative visual acuity analysis

**Fig. 4:**
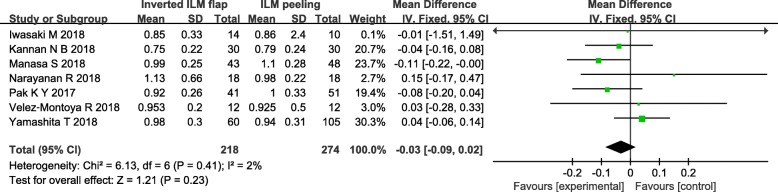
Forest plots of preoperative VA between inverted ILM flap and ILM peeling group. Michalewska’s study was not included in the analysis of preoperative VA due to the SD of preoperative VA was not given

One [9] of the eight trials didn’t report standard deviation (SD) of preoperative VA after vitrectomy, thus the remaining seven trials were included. The forest plots of preoperative VA revealed that the differences were not statistically significant between the inverted ILM flap technique group and ILM peeling group (MD = − 0.03, 95% CI = − 0.09 to 0.02; *P* = 0.23; Fig. 4). There was mild statistical heterogeneity between the studies (heterogeneity I^2^ = 2%), of which influence to the pooled result could be neglected.

### Postoperative visual acuity analysis

**Fig. 5:**
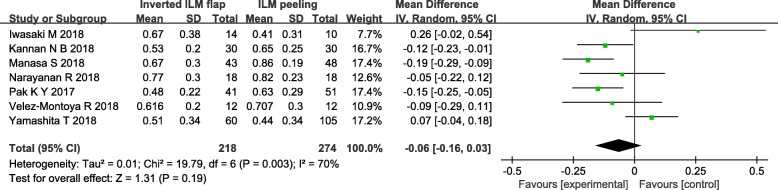
Forest plots of postoperative VA between inverted ILM flap and ILM peeling group. Michalewska’s study was not included in the analysis of postoperative VA due to the SD of postoperative VA was not given

To analysis the results of comparing postoperative VA between the group of inverted ILM flap technique and ILM peeling, we included the seven trials all reported the postoperative VA after surgery except one trial [9] that didn’t mentioned SD of postoperative VA. There was no statistically significant difference in postoperative VA between two groups (MD = − 0.06, 95% CI = − 0.16 to 0.03; *P* = 0.19; Fig. 5). However, Heterogeneity was relatively high (heterogeneity I^2^ = 70%).

**Fig. 6:**
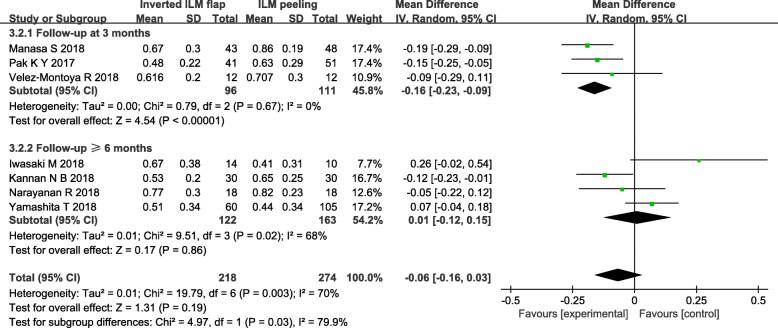
Forest plots of postoperative VA based on follow-up duration in the subgroup analysis. The subgroup analysis based on follow-up duration that divided the included studies into follow-up duration at 3 months and follow-up duration ≥ 6 months. Michalewska’s study was not included

In order to eliminate the effect of follow-up time to the result, We carried out the subgroup analysis based on follow-up duration that divided the included studies into follow-up duration at 3 months and follow-up duration ≥6 months. In the subgroup analysis of follow-up duration at 3 months including 3 studies [4, 10, 12], postoperative VA was significantly better in the group of inverted ILM flap than ILM peeling (MD = − 0.16, 95% CI = − 0.23 to 0.09; *P* < 0.00001; Fig. 6). We observed no heterogeneity between 3 studies in the subgroup of follow-up duration at 3 months (heterogeneity I^2^ = 0%). The analysis of a subgroup with a longer follow-up period did not show any statistically significant differences between the group of inverted ILM flap technique and ILM peeling (MD = 0.01, 95% CI = − 0.12 to 0.15; *P* = 0.86; Fig. 6). Moreover, there was still high heterogeneity between these trials in the subgroup of follow-up duration ≥6 months (heterogeneity I^2^ = 68%). After reading the included four studies [11, 13–15] carefully, a random effects model was used in the later subgroup.

### Publication bias analysis

**Fig. 7:**
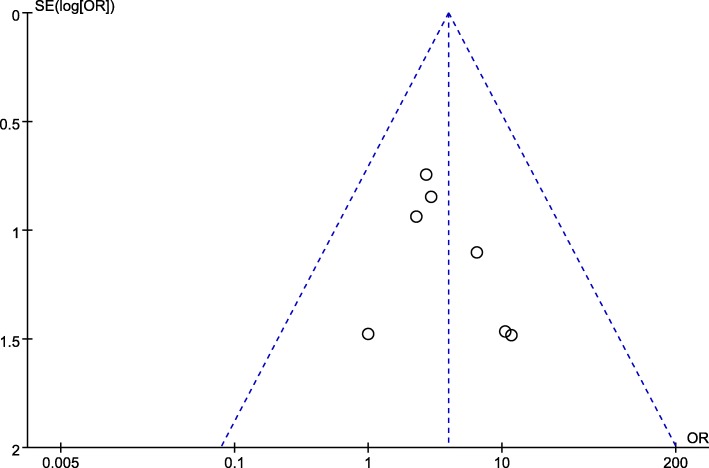
Funnel plot analysis of MH closure rate. SE standard error, OR odds ratio

**Fig. 8:**
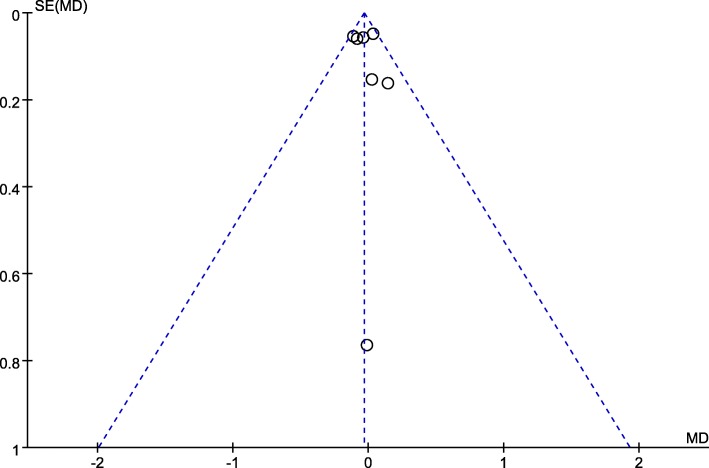
Funnel plot analysis of preoperative VA

Two funnel plots of the rate of MH closure, the preoperative VA showed that the scattered points of the included studies were distributed in the middle and top of the baseline, and most points were located in the range of inverted funnel. It suggested that there was no serious publication bias and the conclusion is relatively reliable (Figs. 7, 8).

## Discussion

We conducted this systematic review and double arm meta-analysis of literature to summarize current evidence and compare the anatomical and visual outcomes of vitrectomy with inverted ILM flap technique and ILM peeling. After pooling the results of the rate of MH closure in these included studies, we performed sensitivity analysis to find the source of heterogeneity. This study [14] was considered to be remove, because its limitations included small sample size, non-standardized measurements, the presence of multiple surgeons, the utilization of multiple OCT machines, and so on. After eliminating the interference of heterogeneity, the result indicated that the rate of MH closure was significantly higher in vitrectomy with inverted ILM flap than that in vitrectomy with ILM peeling.

In histopathologic findings, when the margins of the ILM were left attached to the edges of the hole, it served as a gap-free natural scaffold for gliosis, and provided a basement membrane for proliferateglial glial cells to maintain anatomic structure of the foveola [9, 10]. Shiode et al. showed that the neurotrophic factors and basic fibroblast growth factors (bFGF) on the surface of the ILM flap promoted the proliferation and migration of the Müller cells, contributing to fill the MH and enhance closure [11]. Activated Müller cells also produced neurotrophic factors and growth factors that may promote the survival of retinal neurons [18]. Moreover, the ILM flaps create a closed compartment enabling the RPE to pump out fluid and keep hole dry [10]. These findings explained to the better anatomical results in the inverted ILM flap technique group.

As for the outcomes of visual functions, the preoperative and postoperative VA were observation indexes in this review. There were not significant in the preoperative VA between the two groups, which reduced pre-intervention effects to postoperative VA. Considering the heterogeneity in postoperative VA between these trials, the included studies divided into two subgroups based upon follow-up duration to observe the short and long-term visual efficacy. Compared with ILM peeling, postoperative VA was better in the group of inverted ILM flap technique at the follow-up of 3 months. It seemed that flap technique had a signifcant visual gain in the short term. It was believed that the ILM flaps was also a bridge leading photoreceptor to migrate into the retina defect [19]. Michalewska et al. [9] found that glial cells proliferated and produced an environment to transfer light from the retinal surface to the photoreceptor cell layer. In the cases after the surgery of inverted ILM flap technique, retinal tissue regenerated from the external limiting membrane (ELM), then regrowth of the ellipsoid layer was observed over the next few months [4, 20].

However, contrary to expectation, our results indicated no difference in functional outcomes between the two groups of different surgical treatments at relatively long-term follow-up over 6 months. Hayashi et al. [21] suggested that the photoreceptor layer of fovea might be destroyed and had irreversible damage. Ota et al. [22] showed the fovea losed its original stratified structure in large MH. The IS/OS and ELM would returned gradually after surgery, but not completely or in all cases. The improvement of the foveal structure did not include the restoration of the normal layered structure of retina. There were no significant differences in structural changes over time after inverted ILM flap technique or ILM peeling. Moreover, the differences in the baseline characteristics such as MH size related to the results [14, 22]. These findings might explain the limitations in visual recovery after vitrectomy with inverted ILM flap technique at a longer follow-up.

For all we know, double-arm study to compare the efficacy in large MH after treatments between inverted ILM flap technique and ILM peeling was reviewed and analyzed firstly in this meta-analysis. However, several limitations were inevitable and should be taken into account when citing the results of the meta-analysis. First, the number of included studies and available data were limited. Second, this systematic review included not only RCTs but also retrospective studies, which might have potential sources of selection bias. Third, significant heterogeneity among the studies was detected in the comparison of primary and secondary end points. We alleviated but not eliminated completely the heterogeneity through sensitivity analysis and subgroup analysis. The pooled data were from the relative long-term follow-up durations of the studies, introducing a potential heterogeneity. Follow-up durations in the included trials were not long enough for better observations of VA recovery in long term. Furthermore, other influence factors, such as dye for ILM stained or the specific forms of inverted ILM flap technique among the studies might serve as the points of heterogeneity. Due to the deficiency of these available data, we did not perform subgroup analysis.

## Conclusions

Our meta-analysis indicated that vitrectomy with inverted ILM flap technique had a better anatomical outcome than ILM peeling in large MH. Flap technique had a signifcant visual gain in the short term, but the limitations in visual recovery at a longer follow-up was found. Moreover, there is an urgent need for long follow-up duration and large prospective randomized study to further confirm the efficacy of inverted ILM flap technique and ILM peeling.

## Data Availability

The data that support the findings of this study are available in the reference [4, 9–15].

## Abbreviations

bFGF: Basic fibroblast growth factors
CI: Confidence interval
ELM: External limiting membrane
ILM: Internal limiting membrane
logMAR: Logarithm of minimal angle of resolution
MD: Mean difference
MH: Macular hole
M-H: Mantel–Haenszel
NOS: Newcastle-Ottawa Scale
OR: Odds ratio
RCT: Randomized control trial
SD: Standard deviation
SE: Standard error
VA: Visual acuity
